# Microglial BDNF, PI3K, and p-ERK in the Spinal Cord Are Suppressed by Pulsed Radiofrequency on Dorsal Root Ganglion to Ease SNI-Induced Neuropathic Pain in Rats

**DOI:** 10.1155/2019/5948686

**Published:** 2019-04-28

**Authors:** Xueru Xu, Shaoxiong Fu, Xiaomei Shi, Rongguo Liu

**Affiliations:** Department of Pain Management, Fujian Provincial Hospital, Fujian Key Laboratory of Geriatrics, Provincial Clinic College of Fujian Medical University, Fuzhou, Fujian, China

## Abstract

**Background:**

Pulsed radiofrequency (PRF) on the dorsal root ganglion (DRG) has been applied to alleviate neuropathic pain effectively, yet the mechanisms underlying pain reduction owing to this treatment are not clarified completely. The activated microglia, brain-derived neurotrophic factor (BDNF), phosphatidylinositol 3-kinase (PI3K), and phosphorylated extracellular signal-regulated kinase (p-ERK) in the spinal cord were demonstrated to be involved in developing neuropathic pain. Also, it has been just known that PRF on DRG inhibits the microglial activation in nerve injury rats. Here, we aim to investigate whether PRF treatment could regulate the levels of BDNF, PI3K, and p-ERK in the spinal cord of rats with spared nerve injury (SNI) via suppressing the spinal microglia activation to ease neuropathic pain.

**Methods:**

The rats with SNI were intrathecally treated with minocycline (specific microglia inhibitor) or same volume of dimethyl sulfoxide once daily, beginning from 1 h before nerve transection to 7 days. PRF was applied adjacent to the L_4_-L_5_ DRG of rats with SNI at 45 V for 6 min on the seventh postoperative day, whereas the free-PRF rats were treated without PRF. The withdrawal thresholds were studied, and the spinal levels of ionized calcium-binding adapter molecule 1 (Iba1), BDNF, PI3K, and p-ERK were calculated by western blot analysis, reverse transcription-polymerase chain reaction, and immunofluorescence.

**Results:**

The paw withdrawal mechanical threshold and paw withdrawal thermal latency decreased in the ipsilateral hind paws after SNI, and the spinal levels of Iba1, BDNF, PI3K, and p-ERK increased on day 21 after SNI compared with baseline (*P* < 0.01). An intrathecal injection of minocycline led to the reversal of SNI-induced allodynia and increase in levels of Iba1, BDNF, PI3K, and p-ERK. Withdrawal thresholds recovered partially after a single PRF treatment for 14 days, and SNI-induced microglia hyperactivity, BDNF upregulation, and PI3K and ERK phosphorylation in the spinal cord reduced on D14 due to the PRF procedure.

**Conclusion:**

Microglial BDNF, PI3K, and p-ERK in the spinal cord are suppressed by the therapy of PRF on DRG to ease SNI-induced neuropathic pain in rats.

## 1. Introduction

Neuropathic pain is a kind of refractory pain that arises as a direct consequence of a lesion or disease affecting the somatosensory system [1, 2]. A variety of damages to the peripheral nerves, including diabetes, zoster virus, human immunodeficiency virus-acquired immunodeficiency syndrome, and compression injury, can result in neuropathic pain [3]. Neuropathic pain, characterized by hyperalgesia or allodynia, is associated with central and peripheral sensitization of neurons in the nociceptors [4, 5]. It is hard to treat due to the complicated etiology and mechanisms, including several neurotransmitter systems, receptors, ionic channels, and cell types [6, 7]. Thus, current pharmacotherapy rarely resolves intractable pain in patients. Pulsed radiofrequency (PRF), a type of electromagnetic stimulation, has been successfully used to treat patients suffering from neuropathic pain [8, 9]. PRF on dorsal root ganglion (DRG) is considered to be superior to continuous radiofrequency because the electrode tip temperature of PRF does not exceed 42°C during the whole process to avoid massive tissue destruction. Nowadays, the application of PRF on DRG to treat neuropathic pain has greatly helped clinicians. However, the analgesic mechanism of this therapy is not well clarified so far.

Recently, PRF was administered on DRG in rats with peripheral nerve injury (PNI) to downregulate microglial activation in the spinal cord and improve pain behaviors [10, 11]. Microglia are the resident macrophages in the central nervous system (CNS), and they react to the stimuli that may affect homeostasis and induce pathological alterations [12]. As a consequence of multiple types of damages in the nervous system, microglia can transform to reactive states through a progressive series of cellular and molecular changes, including morphological hypertrophy, proliferation, and expression of various genes [13]. The activated microglial cells play a key role in the peripheral and central sensitization to develop neuropathic pain conditions [14]. They secrete brain-derived neurotrophic factor (BDNF), which is a critical microglia-neuron signaling molecule that gates aberrant nociceptive processing in the spinal cord [15]. Many studies support the pronociceptive role of BDNF in pain processes in the peripheral and CNS. Nociceptor-derived BDNF has been shown to be involved in inflammatory pain and microglial-derived BDNF in neuropathic pain [16]. Recently, Liu et al. [17] reported that BDNF participated in colitis-induced spinal central sensitization, and the phosphatidylinositol 3-kinase (PI3K)/protein kinase B pathway mediated BDNF action in the spinal cord. Moreover, the second messengers that PI3K generated could activate phosphorylated extracellular signal-regulated kinase (p-ERK) [18, 19]. In microglia, ERK activation occurred after nerve injury, and the inhibition of the activated ERK could suppress neuropathic pain development [20]. The spinal cord, which is the primary integration center of information and plays a crucial role in central sensitization, was preferred by pain physicians for exerting neuromodulation to relieve neuropathic pain, such as spinal cord stimulation. In precedent different animal models, the microglia, BDNF, PI3K, and p-ERK were involved in the development of neuropathic pain. However, it remains uncertain that whether the release of BDNF, PI3K and p-ERK in the spinal cord regulates chronic pain processing through microglia-dependent mechanism. In addition, whether the application of PRF on DRG for treating neuropathic pain is associated with the downregulated levels of microglia, BDNF, PI3K, and p-ERK in the spinal cord needs exploration further.

In this study, a microglial inhibitor was intrathecally administered to the rats with spared nerve injury (SNI) to affirm the release of BDNF, PI3K, and p-ERK in the spinal cord via the microglial-dependent mechanism. We also investigated whether PRF treatment could regulate the levels of BDNF, PI3K, and p-ERK in the spinal cord of SNI rats via suppressing the spinal microglia activation to alleviate the neuropathic pain.

## 2. Materials and Methods

### 2.1. Animals

Male Sprague–Dawley (SD) rats (4-month-old, 250–280 g) were obtained from the Experimental Animal Center of Fujian Medical University, Fuzhou. The animals were housed under a 12 h light-dark cycle at 22°C–24°C with ad libitum access to food and water in the Pharmacy College of Fujian Medical University (SPF class). All procedures in this study were approved by the Fujian Medical University Experimental Animal Welfare Ethics Committee (SYXK 2016-0007).

### 2.2. Treatment Group and Design

Ninety male SD rats were randomly (according to the method of random number table) divided into six groups (*n* = 15, each): Sham group, SNI group, SNI + PRF group, SNI + free-PRF group, SNI with minocycline (SNI + M) group, and SNI with dimethyl sulfoxide (SNI + DMSO) group.

#### 2.2.1. Effect of Minocycline on Levels of Microglia, BDNF, PI3K, and ERK in the Spinal Cord

The rats in the following four groups (Sham, SNI, SNI + M, and SNI + DMSO) were studied. All rats (except the Sham group) were subjected to SNI of the right sciatic nerve. The rats in the SNI + M and SNI + DMSO groups were intrathecally treated with minocycline (specific microglia inhibitor) and an equal volume of DMSO, respectively. Pain behaviors and the levels of ionized calcium-binding adapter molecule 1 (Iba1), BDNF, PI3K, and p-ERK in the spinal cord were assayed and compared among the four groups.

#### 2.2.2. Effect of PRF on DRG on the Neuropathic Pain and Levels of Microglia, BDNF, PI3K, and ERK in the Spinal Cord

Four groups were observed, including Sham, SNI, SNI + PRF, and SNI + free-PRF. All rats (except those in the Sham group) were subjected to SNI of the right sciatic nerve. On postoperative day 7, PRF was applied to the ipsilateral L_4_-L_5_ DRG in the SNI + PRF group, and SNI + free-PRF group was kept as a control. Pain behaviors and levels of Iba1, BDNF, PI3K, and p-ERK were measured.

The von Frey behavioral testing for paw withdrawal mechanical threshold (PWMT) and sting thermal imaging for paw withdrawal thermal latency (PWTL) were performed before the operation (D0), on 1st (D01), 3rd (D03), 5th (D05), and 7th (D07) postoperative days, and 1st (D1), 3rd (D3), 5th (D5), 7th (D7), 9th (D9), 11th (D11), and 14th (D14) days after PRF treatment or completion of intrathecal injection. The expression levels of Iba1, BDNF, PI3K, and p-ERK in the spinal cord were measured by western blot analysis, reverse transcription-polymerase chain reaction (RT-PCR), and immunofluorescence on D14. Western blot, RT-PCR, and immunofluorescence were applied to five rats each.

### 2.3. Neuropathic Pain Model

PNI was performed according to the SNI model as described by Decosterd and Woolf [21]. Briefly, the rat's right common sciatic nerve at the trifurcation into the tibial, common peroneal, and sural nerves of the rats was exposed under anesthesia. The tibial and common peroneal nerves were transected, leaving the sural nerve intact. The procedures were performed in the same way without transecting the nerves in the Sham group. Then, the muscles were massaged back into place, and the incision was closed. All operations were performed by the same researcher.

### 2.4. Intrathecal Catheters and Drug Administration

The rats received intrathecal catheter implantation before SNI. They were anesthetized with an intraperitoneal injection of 10% chloral hydrate (300 mg/kg). A PE-10 polyethylene catheter was implanted between the L_5_ and L_6_ vertebrae to reach the subarachnoid space of the spinal cord as described in a previous study [22]. The outer part of the catheter was plugged and fixed onto the skin upon wound closure. The rats showing neurological deficits after the catheter implantation were euthanized. Minocycline was dissolved in sterile 5% DMSO with 95% saline solution. The rats were intrathecally treated with minocycline (40 mg/kg, Sigma-Aldrich, St. Louis, MO, USA) or an equivalent volume of DMSO once daily for 7 days, 1 h before nerve transection. Drugs or vehicles were intrathecally injected through the implanted catheter in a 10 *μ*l volume of solution followed by 10 *μ*l of vehicle for flushing. Each injection lasted for at least 5 minutes. After injection, the needle was retained *in situ* for 2 minutes before being withdrawn.

### 2.5. PRF on DRG

The rats received PRF treatment on day 7 after SNI. They were anesthetized using an injection of 10% chloral hydrate (300 mg/kg). The right L_4_-L_5_ DRG was exposed in the SNI + free-PRF and SNI + PRF groups through laminectomy and facetectomy, without injury to the dura mater. An RF electrode (type 20 G, 5 cm long, 4 mm active tip) was placed adjacent to the corresponding DRG via direct visualization by using a radiofrequency device (Cosman Medical, Inc., Burlington, MA, USA) The motor stimulation test was used instead of the sensory stimulation test. PRF waves were applied after carrying out the motor stimulation test through muscle contraction of the lower extremities. Stimulation parameters of the PRF waves were set as follows: 2 bursts/s; duration = 20 ms; output voltage = 45 V; maximum temperature = 42°C; and the stimulated time = 6 min. After the PRF treatment, the RF probe was removed, and the muscles were closed. In the SNI + free-PRF group, the electrode was put in the same way without any stimuli.

### 2.6. Behavioral Testing

The rats were placed in a plastic chamber (20 × 25 × 15 cm^3^) and habituated for 15 min before the experiment. PWMT was evaluated using von Frey filaments (Stoelting, IL, USA) by the up-down method described in a previous study [23]. Each filament was applied perpendicularly to the ipsilateral territory, near the center of the vibrissal pad. Avoiding further contact with the filament, quickly turning head away, scratching the stimulated area, or attacking the filament was considered a positive response. An allodynic rat was defined as the one with 50% PWT <4.0 g (withdrawal in response to nonnoxious tactile stimulus).

PWTL was tested by measuring the withdrawal response of the hind paw to heat stimulation using the Plantar Test Apparatus (TaiMeng Science and Technology, Chengdu, China) as described by Hargreaves et al. [24]. The cutoff latency was 30 s to avoid thermal injury. The withdrawal latency at each time point was an average of three latencies separated by an interval of 5 min. The tests were conducted on the same days as the von Frey test, and both tests were conducted by the same researcher who was blind to the group allocation of the rats.

### 2.7. Western Blot Analysis

The spinal cord of the lumbar (L_4_-L_5_) ipsilateral quadrant to the lesion was collected, dissected, and homogenized in protein lysis buffer in the presence of protease inhibitors and incubated on ice for 10 min. The samples were centrifuged at 12,000 rpm for 15 min at 4°C. The total protein content was determined in the supernatants using the Bio-Rad DC Protein Assay Kit. Equal amounts of protein were resolved by 10% SDS-PAGE and transferred to PVDF membranes (Millipore, MA, USA). The membranes were blocked with 5% nonfat milk at room temperature and incubated overnight at 4°C with primary antibodies (rabbit anti-Iba1, ab178680, 1 : 1000, Abcam, USA; rabbit anti-BDNF, ab108319, 1 : 1000, Abcam, USA; rabbit anti-p-ERK, #4370, 1 : 1000, Cell Signaling Technology, USA; rabbit anti-PI3K, ab40776, 1 : 2000, Abcam, USA). Then, the membranes were incubated with a horse-radish peroxidase-conjugated secondary antibody (1 : 5000, Thermo Scientific, USA) at room temperature for 2 h. Finally, peroxidase activity was visualized using the ECL Western Blot Detection Kit (Beyotime, China). Western blots were quantitated using an image analysis system (Bio-Rad, USA). After normalization with *β*-actin, the data were presented as mean percentages of the ratio of total protein to their respective signal intensity levels found in the Sham group animals, indicated as 100%.

### 2.8. Real-Time RT-PCR

The total RNAs were extracted from the L_4_-L_5_ ipsilateral quadrant spinal cord using TRIzol and reverse transcribed using the High-Capacity cDNA Reverse Transcription Kit (Applied Biosystems, CA, USA). The real-time PCR was performed using the Power SYBR Green Master Mix (Applied Biosystems) according to the manufacturer's protocols and analyzed by RT-PCR in a detection system (Applied Biosystems). The real-time PCR protocol was as follows: reverse transcriptase was activated and cDNA was synthesized (50°C for 5 min), PCR was activated (95°C for 3 min), 40 cycles of denaturation were performed (95°C for 30 s), and annealing and extension were done for 1 min at 60°C. At the end of PCR, a melting curve analysis was performed by slowly increasing the temperature from 60°C to 95°C. The data were analyzed using Software 2.2 using a cycle threshold (Ct) value as the readout and relatively normal levels of *β*-actin.

The primers used were as follows:

  Iba1: 5′-GCAAGGATTTGCAGGGAGGA-3′ (forward), 5′-TGGGATCATCGAGGAAGTGC-3′ (reverse)
  BDNF: 5′-AATAATGTCTGACCCCAGTGCC-3′ (forward), 5′-CTGAGGGAACCCGGTCTCAT-3′ (reverse)
  PI3K: 5′-ATTTCCAGTGGGTGAGGCAG-3′ (forward), 5′-CTCATGGTAGCCGGTGACTC-3′ (reverse)
  p-ERK: 5′-ATTATGTGCACCGGGACCTG-3′ (forward), 5′-TGTCATCCTGGAGGTAGCGA-3′ (reverse)
 
*β*-Actin: 5′-ACTCTGTGTGGATTGGTGGC-3′(forward), 5′-AGAAAGGGTGTAAAACGCAGC-3′(reverse)

### 2.9. Immunofluorescence Histochemistry

The rats were perfused with 200 mL of saline followed by 200 mL of 0.1 M phosphate buffer (pH 7.3) containing 4% paraformaldehyde. The L_4_-L_5_ spinal cord was removed, postfixed in 4% paraformaldehyde for 24 h, and allowed to equilibrate in 30% sucrose in phosphate-buffered saline (PBS) overnight at 4°C. Transverse spinal sections (4–6 *μ*m) were cut using a cryostat and collected in 0.01 M PBS, pH 7.3. After washing with PBS, the tissue was penetrated with 0.3% Triton X-100 and primary antibodies for rabbit anti-rat Iba1, BDNF, p-ERK, and PI3K (rabbit anti-Iba1, ab178680, 1 : 100, Abcam, USA; rabbit anti-BDNF, ab108319, 1 : 500, Abcam, USA; rabbit anti-p-ERK, #4370, 1 : 200, Cell Signaling Technology, USA; rabbit anti-PI3K, ab40776, 1 : 200, Abcam, USA). Next, the slides were covered with secondary antibodies containing 1 *μ*M 4′-6-diamidino-2-phenylinedole (Sigma, USA). Some sections stained for BDNF, p-ERK, and PI3K were double labeled using cell-type-specific Abs for microglia (Iba1). The nuclei were stained with DAPI (5 *μ*g/mL; Beyotime, USA). Fluorescence signal was detected using a fluorescence microscope (Olympus, Japan), images were captured, and signal co-localization was measured using MetaMorph (Molecular Devices, USA). The area fraction was quantified using Image J software (Rawak Software, Inc., Germany).

## 3. Statistical Analysis

All data were analyzed using SPSS 20.0 statistical software package (SPSS Inc., IL, USA) and presented as mean ± standard error of mean (SEM). All data were graphed using Prism 5.0 (GraphPad, CA, USA). After the data distribution was tested to be normal, behavioral data, western blot data, and enzyme-linked immunosorbent assay data were analyzed using a repeated-measures (multiple groups × time) analysis of variance (ANOVA). Multiple comparisons were performed using the Bonferroni post hoc test to determine the overall significance. When ANOVA showed a significant difference, pairwise comparisons between the means were tested using the post hoc Tukey method or Fisher's protected least significant difference (LSD) post hoc test. An alpha value of 0.05 was considered statistically significant.

## 4. Results

### 4.1. Inhibiting the Spinal Microglia Activation Produced a Significantly Neuropathic Pain Reduction in SNI Rats

**Figure 1:**
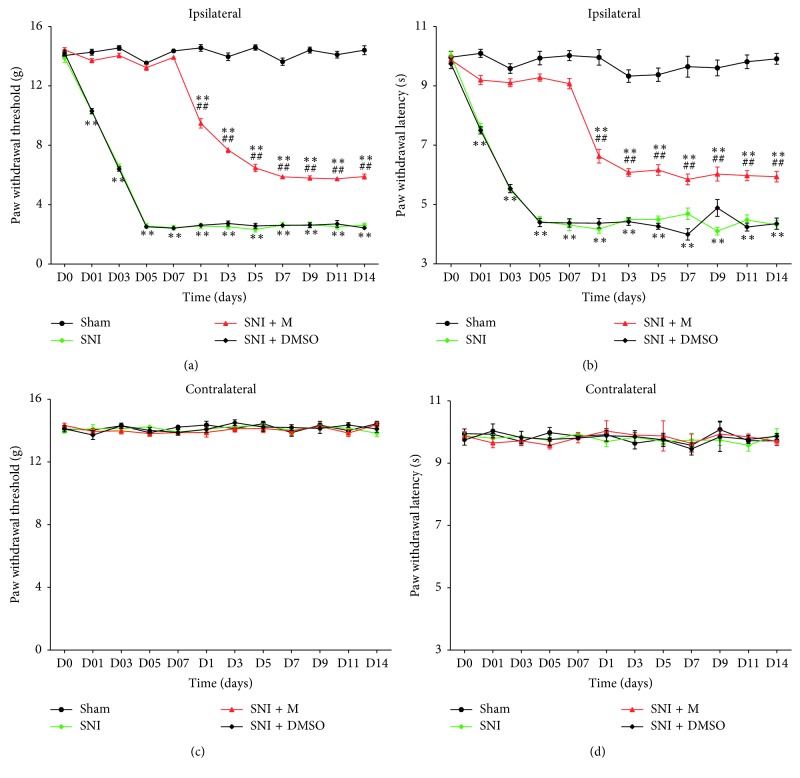
Therapeutic effects of minocycline on neuropathic pain in SNI rats. (a, b) Effects of minocycline (a specific inhibitor of microglial activation) on mechanical allodynia and thermal hyperalgesia. Reversal of SNI-induced allodynia by intrathecal administration of minocycline once a day for 7 days (1 h before nerve ligation) in the rats. All rats (except those in the Sham group) were subjected to SNI of the right sciatic nerve. The rats in the SNI + M and SNI + DMSO groups were intrathecally treated with minocycline and an equal volume of DMSO, respectively. Each symbol represents mean ± SEM; ^*∗∗*^*P* < 0.01 against the Sham group and ^##^*P* < 0.01 against the SNI group. Repeated-measures (multiple groups × time) ANOVA, *n* = 15 per group. (c, d) Changes in paw withdrawal threshold and paw withdrawal latency in contralateral hind paw of all groups.

Compared with baseline, the SNI group displayed long-lasting mechanical allodynia (*P* < 0.01; Figure 1(a)) and thermal hyperalgesia (*P* < 0.01; Figure 1(b)) in their ipsilateral paws, which reached a peak on the fifth day and maintained stable withdrawal thresholds until the end of observation. No significant changes were found in the contralateral hind paw in all the groups (*P* > 0.05; Figures 1(c) and 1(d)), similar to the ipsilateral paw in the Sham group (*P* > 0.05; Figures 1(a) and 1(b)). The mechanical allodynia and thermal hyperalgesia were not induced during intrathecal injection of minocycline. Although the withdrawal thresholds significantly decreased after completing injections in the SNI + M group compared with those in the Sham group (*P* < 0.01; Figures 1(a) and 1(b)), they were still higher than the values in the SNI and SNI + DMSO groups (*P* < 0.01; Figures 1(a) and 1(b)). No significant differences were found between the SNI and SNI + DMSO groups (*P* > 0.05; Figures 1(a) and 1(b)).

### 4.2. Suppression of Microglia Activation Contributes to a Remarkable Reduction of the Expression of Iba1, BDNF, PI3K, and p-ERK in the Spinal Cord

**Figure 2:**
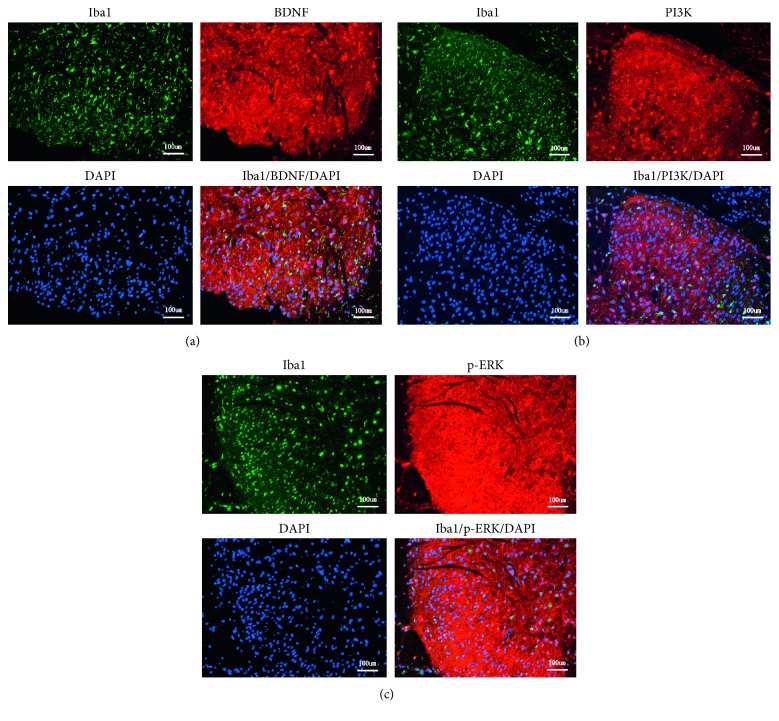
Three immunolabeling of Iba1/DAPI with BDNF (a), PI3K (b), and p-ERK (c). At a higher magnification, almost all BDNF, PI3K, and p-ERK immunofluorescence colocalized with the nuclear marker DAPI. The localization of BDNF, PI3K, and p-ERK in microglia was confirmed by triple labeling with BDNF/Iba1/DAPI, PI3K/Iba1/DAPI, and p-ERK/Iba1/DAPI. Scale bar: 100 *μ*m.

**Figure 3:**
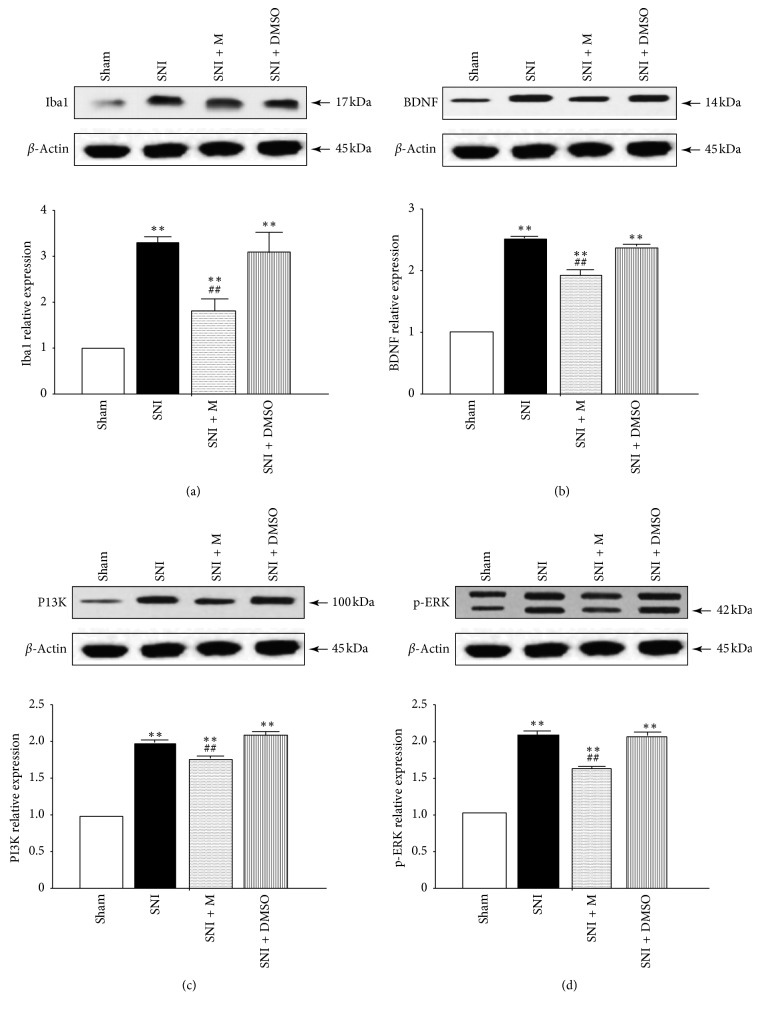
The western blot analysis of Iba1 (a), BDNF (b), PI3K (c), and p-ERK (d) proteins in the spinal cord of rats in different groups on D14. Values represented the relative ratio of Iba1, BDNF, PI3K, or p-ERK levels (normalized to *β*-actin) to that in the Sham rats. Each symbol represents mean ± SEM. In the Iba1 assay, ^*∗∗*^*P* < 0.01 against the Sham group and ^##^*P* < 0.01 against the SNI group. In the BDNF assay, ^*∗∗*^*P* < 0.01 against the Sham group and ^##^*P* < 0.01 against the SNI group. In the PI3K assay, ^*∗∗*^*P* < 0.01 against the Sham group and ^##^*P* < 0.01 against the SNI group. In the p-ERK assay, ^*∗∗*^*P* < 0.01 against the Sham group and ^##^*P* < 0.01 against the SNI group. LSD *t* test, *n* = 5 rats per assay.

**Figure 4:**
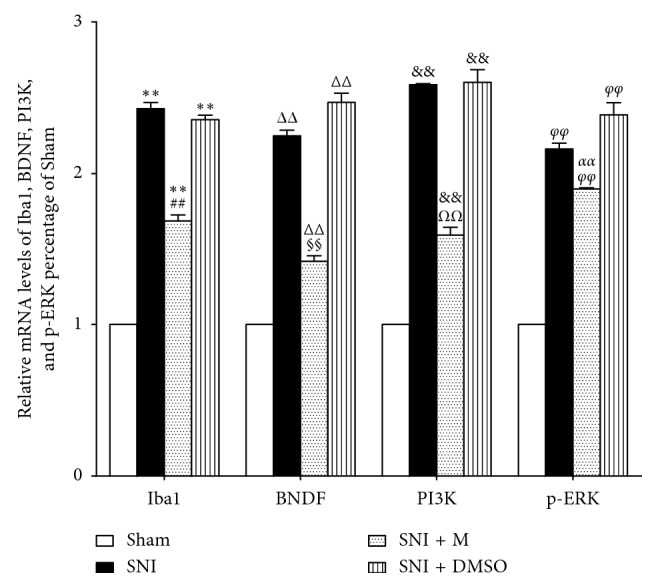
Real-time PCR analysis of Iba1, BDNF, PI3K, and p-ERK mRNA expression in the spinal cord in different groups on D14. Values represent the relative ratio of Iba1, BDNF, PI3K, and p-ERK mRNA (normalized to GAPDH mRNA) expression to that in the Sham rats. Each symbol represents mean ± SEM. In the Iba1 assay, ^*∗∗*^*P* < 0.01 against the Sham group and ^##^*P* < 0.01 against the SNI group. In the BDNF assay, ^ΔΔ^*P* < 0.01 against the Sham group and ^§§^*P* < 0.01 against the SNI group. In the PI3K assay, ^&&^*P* < 0.01 against the Sham group and ^*ΩΩ*^*P* < 0.01 against the SNI group. In the p-ERK assay, ^*φφ*^*P* < 0.01 against the Sham group and ^*αα*^*P* < 0.01 against the SNI group. LSD *t* test, *n* = 5 rats per assay.

**Figure 5:**
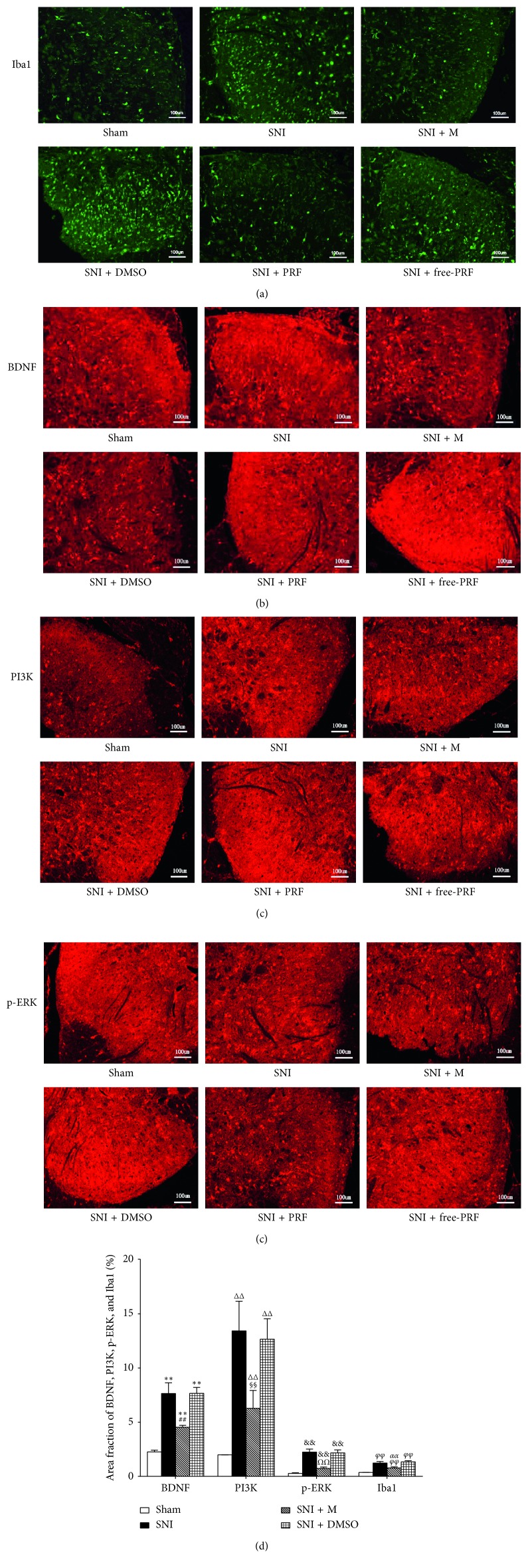
Expression of Iba1 (a), BDNF (b), PI3K (c), and p-ERK (d) in the spinal cord of rats in different groups (immunofluorescence, ×100), scale bar = 100 *μ*m. (e) The intensity of Iba1, BDNF, PI3K, and p-ERK immunofluorescence in the spinal cord in different groups on D14. Each symbol represents mean ± SEM. In the BDNF assay, ^*∗∗*^*P* < 0.01 against the Sham group and ^##^*P* < 0.01 against the SNI group. In the PI3K assay, ^ΔΔ^*P* < 0.01 against the Sham group and ^§§^*P* < 0.01 against the SNI group. In the p-ERK assay, ^&&^*P* < 0.01 against the Sham group and ^*ΩΩ*^*P* < 0.01 against the SNI group. In the Iba1 assay, ^*φφ*^*P* < 0.01 against the Sham group and ^*αα*^*P* < 0.01 against the SNI group. LSD *t* test, *n* = 5 rats per assay.

At a higher magnification, almost all BDNF, PI3K, and p-ERK immunofluorescence co-localized with the nuclear marker DAPI (Figures 2(a)–2(c)). The localization of BDNF, PI3K, and p-ERK in microglia was confirmed by triple labeling with BDNF/Iba1/DAPI, PI3K/Iba1/DAPI, and p-ERK/Iba1/DAPI (Figures 2(a)–2(c)). Western blot analysis (Figures 3(a)–3(d)), RT-PCR analysis (Figure 4), and immunofluorescence and histochemical analysis (Figures 5(a)–5(e)) in the spinal cord demonstrated that the levels of Iba1, BDNF, PI3K, and p-ERK were low in the Sham group and increased in the ipsilateral spinal cord in the SNI and SNI + DMSO groups (*P* < 0.01). They decreased in the SNI + M group compared with the SNI group but were still higher than those in the Sham group on D14 (*P* < 0.01).

### 4.3. PRF Treatment Results in a Significant Neuropathic Pain Reduction

**Figure 6:**
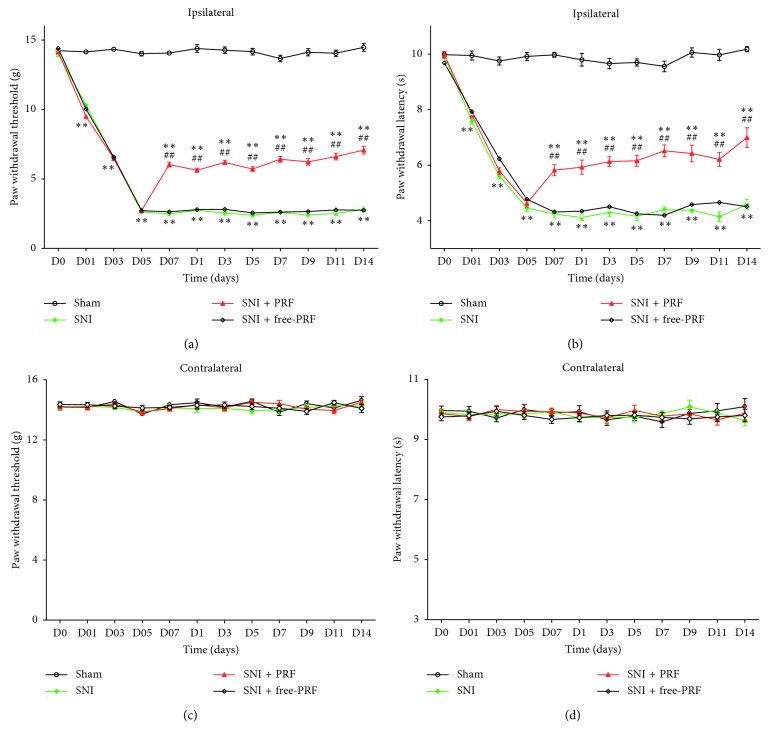
Therapeutic effects of PRF on DRG on neuropathic pain. (a, b) Effects of PRF on mechanical allodynia and thermal hyperalgesia applied to L_4_-L_5_ DRG. The paw withdrawal threshold in response to mechanical hypersensitivity (a) and paw withdrawal latency in response to thermal hyperalgesia (b) partially recovered from the first day after a single application of PRF and maintained throughout a period of 14 days. On postoperative day 7, PRF was applied to the ipsilateral L_4_-L_5_ DRG in the SNI + PRF group, and SNI + free-PRF group was kept as control. Each symbol represents mean ± SEM; ^*∗∗*^*P* < 0.01 against the Sham group and ^##^*P* < 0.01 against the SNI group. Repeated-measures (multiple groups × time) ANOVA, *n* = 15 per group. (c, d) Changes in paw withdrawal threshold and paw withdrawal latency in contralateral hind paw of all groups.

Indeed, PNI induced long-lasting mechanical allodynia (*P* < 0.01; Figure 6(a)) and thermal hyperalgesia (*P* < 0.01; Figure 6(b)) since the first day after SNI, reached a peak on the fifth day, and maintained stable withdrawal thresholds until the end of observation compared with those in the Sham-operated rats. No significant changes were observed in the contralateral hind paw (*P* > 0.05; Figures 6(c) and 6(d)) throughout the duration of the study. Similar results were obtained on the ipsilateral side in the Sham group (*P* > 0.05; Figures 6(a) and 6(b)). In this study, PRF was applied on the L_4_-L_5_ DRG in rats with SNI for 6 min on the seventh day after nerve ligation; mechanical allodynia (*P* < 0.01; Figure 6(a)) and thermal hyperalgesia (*P* < 0.01; Figure 6(b)) were partially recovered in the SNI + PRF group from the first day after a single application of PRF and maintained throughout a period of 14 days, compared with those in the SNI and SNI + free-PRF groups, but could not return to pre-SNI baseline. In the SNI and SNI + free-PRF groups, the paw withdrawal threshold and paw withdrawal latency were maintained at a low level from postlesion 7–21 days.

### 4.4. PRF Treatment Reduced the Levels of Spinal Iba1, BDNF, PI3K, and p-ERK in SNI Rats

**Figure 7:**
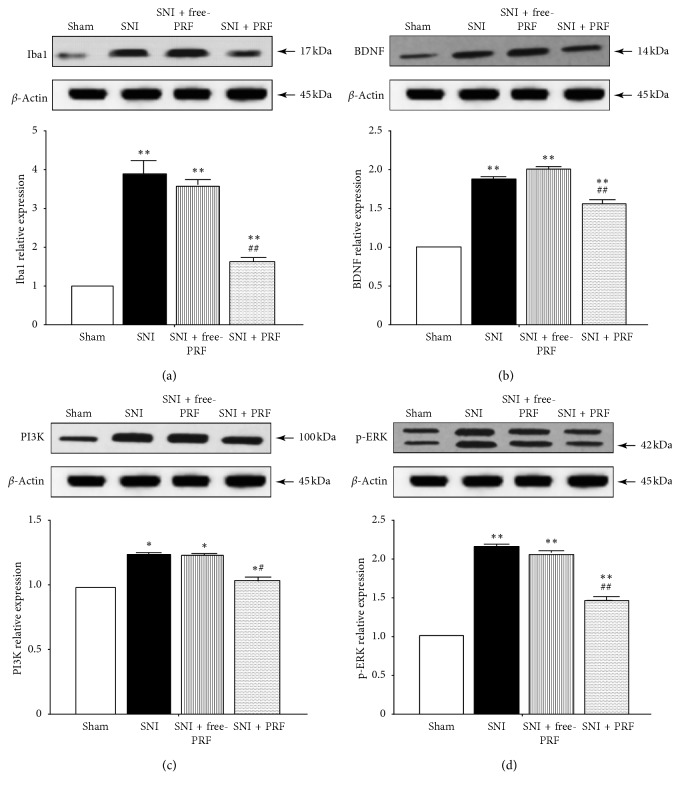
(a–d) The western blot analysis of Iba1, BDNF, PI3K, and p-ERK proteins in the spinal cord of rats in different groups on D14. Values represent the relative ratio of Iba1, BDNF, PI3K, and p-ERK levels (normalized to *β*-actin) to that in the Sham rats. Each symbol represents mean ± SEM. In the Iba1 assay, ^*∗∗*^*P* < 0.01 against the Sham group and ^##^*P* < 0.01 against the SNI group. In the BDNF assay, ^*∗∗*^*P* < 0.01 against the Sham group and ^##^*P* < 0.01 against the SNI group. In the PI3K assay, ^*∗*^*P* < 0.05 against the Sham group and ^#^*P* < 0.05 against the SNI group. In the p-ERK assay, ^*∗∗*^*P* < 0.01 against the Sham group and ^##^*P* < 0.01 against the SNI group. LSD *t* test, *n* = 5 rats per assay.

**Figure 8:**
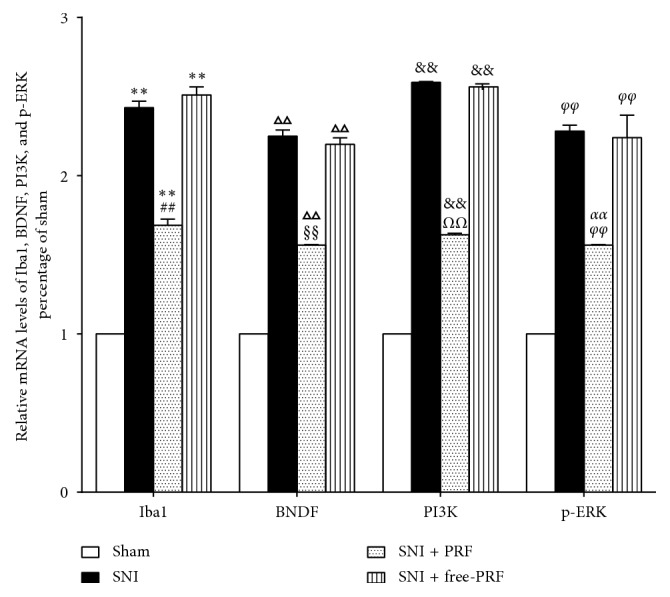
Real-time PCR analysis of Iba1, BDNF, PI3K, and p-ERK mRNA in the spinal cord in different groups on D14. Values represent the relative ratio of Iba1, BDNF, PI3K, and p-ERK mRNA (normalized to GAPDH mRNA) to that in the Sham rats. Each symbol represents mean ± SEM. In the Iba1 assay, ^*∗∗*^*P* < 0.01 against the Sham group and ^##^*P* < 0.01 against the SNI group. In the BDNF assay, ^ΔΔ^*P* < 0.01 against the Sham group and ^§§^*P* < 0.01 against the SNI group. In the PI3K assay, ^&&^*P* < 0.01 against the Sham group and ^*ΩΩ*^*P* < 0.01 against the SNI group. In the p-ERK assay, ^*φφ*^*P* < 0.01 against the Sham group and ^*αα*^*P* < 0.01 against the SNI group. LSD *t* test, *n* = 5 rats per assay.

**Figure 9:**
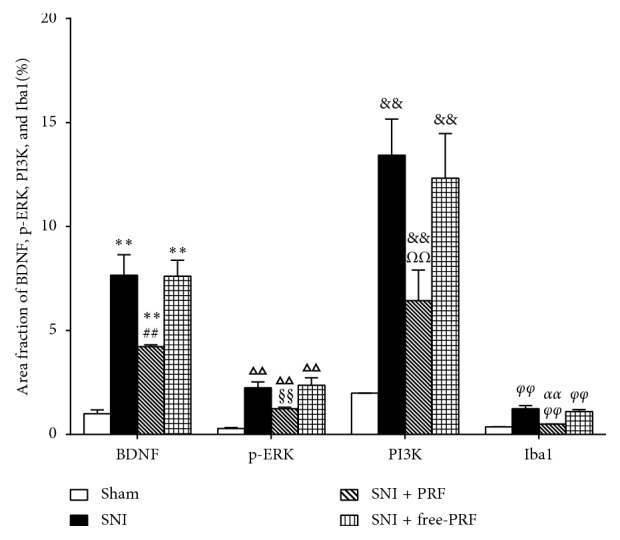
The intensity of Iba1, BDNF, PI3K, and p-ERK immunofluorescence in the spinal cord in different groups on D14. Each symbol represents mean ± SEM. In the BDNF assay, ^*∗∗*^*P* < 0.01 against the Sham group and ^##^*P* < 0.01 against the SNI group. In the p-ERK assay, ^ΔΔ^*P* < 0.01 against the Sham group and ^§§^*P* < 0.01 against the SNI group. In the PI3K assay, ^&&^*P* < 0.01 against the Sham group and ^*ΩΩ*^*P* < 0.01 against the SNI group. In the Iba1 assay, ^*φφ*^*P* < 0.01 against the Sham group and ^*αα*^*P* < 0.01 against the SNI group. LSD *t* test, *n* = 5 rats per assay.

Western blot analysis (Figures 7(a)–7(d)), RT-PCR analysis (Figure 8), and immunofluorescent and histochemical analysis (Figures 5(a)–5(d), and 9) in the spinal cord showed that the expression levels of Iba1, BDNF, PI3K, and p-ERK in the SNI and SNI + free-PRF groups significantly increased compared with those in the Sham group after nerve injury (*P* < 0.05). The levels of Iba1, BDNF, PI3K, and p-ERK in the SNI + PRF group were downregulated significantly than those in the SNI and SNI + free-PRF groups (*P* < 0.05) but were still higher than those in the Sham group after a single application of PRF on D14 (*P* < 0.05).

## 5. Discussion

In the present study, SNI induced a long-lasting increase in microglia hyperactivity and BDNF, PI3K, and p-ERK upregulation in the spinal cord and resulted in pain sensitization. Minocycline, which was intrathecally injected in the early phase of pain generation, relieved mechanical allodynia and thermal hyperalgesia and reversed the upregulated levels of Iba1, BDNF, PI3K, and p-ERK in the spinal cord after injecting interruption. PRF was applied on the ipsilateral DRG of the rats on the seventh day after SNI, which also reversed mechanical allodynia and thermal hyperalgesia. The benefits of a single PRF application persisted for at least 2 weeks after treating interruption. Just like a specific microglia inhibitor, PRF reversed the microglial activation and expression of BDNF, PI3K, and p-ERK in the spinal cord as well.

### 5.1. Behavioral Changes Induced by the Therapy of PRF on DRG

Neuropathic pain is a kind of refractory pain. The conventional painkillers, such as opioids and nonsteroidal anti-inflammatory drugs afford poor efficacy and produce many side effects. Therefore, a good effective therapy for neuropathic pain is urgently required. PRF has been used to treat neuropathic pain and has shown satisfactory efficacy with minimal side effects. PRF electromagnetic pulse conveyed a signal to the target tissue with radiofrequency electrodes of 20 ms, 500 kHz radiofrequency electromagnetic energy, followed by 480 ms interval. The temperature of the tissue around the tip of the PRF does not exceed 42°C to avoid target tissue and nerve damage. DRG is the oval inflation of the dorsal root in the upper region of the intervertebral foramen, which contains the first-class neurons of sensory afferents. DRG has an important role in the process of peripheral sensitization. Nociceptive sensory neurons of DRG are activated by noxious stimuli in the periphery and transmit information to the CNS. The activation of immune and immune-like glial cells in the DRG and spinal cord leads to the release of both pro- and anti-inflammatory cytokines, which are involved in the spinal nociceptive transmission and central sensitization [25, 26]. Because of its active role in the modulation of sensory processing and its anatomic accessibility to clinical intervention, DRG has become an excellent clinical target for pain treatment.

The therapy of PRF on DRG has been demonstrated to improve pain effectively. For example, Arai reported that the PRF on DRG provided pain relief for patients with intractable vertebral metastatic pain [27]. Van Boxem et al. achieved a similar efficacy in chronic intractable lumbosacral radicular pain with PRF therapy on DRG [28]. Here, PRF was applied on DRG to treat neuropathic pain induced by SNI. As neuropathic pain developed significantly and reached the peak point on day 7 after SNI [29], day 7 was appointed as the PRF treatment time in the present study.

Various experimental neuropathic pain models have also been shown a pain-relieving effect of PRF on mechanical hypersensitivity and sometimes on thermal allodynia. The effect of PRF on chronic pain was still very discrepant, likely because of differences in therapy protocols, various exposure time, duration of pain, stimulation site of PRF, pain model, and species used. Treatment with 5 min PRF stimulation on L_5_ DRG in the unilateral L_5_ spinal nerve ligation (SNL) model of rats significantly reduced mechanical hypersensitivity and heat analgesia [30]. In the CFA-induced peripheral inflammatory pain, PRF to the L_4_ anterior primary ramus just close to DRG significantly increased PWMT and PWTL, but PRF to the sciatic nerve in the midthigh just increased PWTL [31, 32]. However, these studies focused on the prophylactic effect of PRF rather than on its therapeutic application for an established chronic pain syndrome. They showed that PRF relieved, even reversed, mechanical hypersensitivity and some thermal allodynia. However, the pain-relief effect was seen on mechanical hypersensitivity only when PRF was applied to established chronic pain [10, 33]. An important finding in the study by Tanaka et al. [34] was that increased exposure time of 2–6 min to PRF current showed a significant antiallodynic effect without motor impairment. Therefore, this study applied PRF current for 6 min to the L_4_-L_5_ DRG in rats with SNI on the seventh day after nerve ligation. PWMT and PWTL were significantly increased following PRF on DRG therapy on day 7 after SNI until day 21. These results were consistent with clinical observations and the findings of Liu et al. [35], further indicating that PRF on DRG was a beneficial treatment for neuropathic pain. A significant finding in this study was that thermal hyperalgesia was also restrained when PRF was applied for established chronic pain. We also observed that nerve injury-induced mechanical allodynia and thermal hyperalgesia could be reversed for long-lasting relief by a single PRF on DRG even after PRF cessation, and they could not return to pre-SNI baseline. The exact mechanisms underlying the analgesic effect of PRF on neuropathic pain deserve further study.

### 5.2. Mechanisms Underlying the Analgesic Effect of PRF

#### 5.2.1. Decreased Microglia Activation

Increasing evidence demonstrates the pivotal role of spinal microglia in neuropathic pain [36, 37]. Following varied types of insults in the nervous system, including PNI, microglial cells were the first to become activated and remained so for several weeks. Microglia transformed to reactive phenotype via displaying a progressive series of cellular and molecular changes, including morphological hypertrophy, rapid proliferation, upregulated expression of various genes, and increased expression of microglia characteristic markers, such as ionized calcium-binding adapter molecule 1 (Iba1). Then, inflammatory cytokines were released by microglia and contributed to the development of pain hypersensitization and long-persisting pain [38]. Inhibitors of microglia by intrathecal administration have shown great analgesic efficacy in pain models [39, 40], but it was limited to reduce the established late-phase pain [41]. In this study, minocycline was intrathecally injected to rats with SNI at early stages. The pain was completely inhibited by minocycline during the period of drug administration. However, the withdrawal thresholds decreased slightly after treatment interruption, and the analgesic effect persisted for 21 days. Moreover, the spinal microglia level was restrained. Our finding also indicated that microglia inhibition as early as possible could gain more long-lasting pain relief.

The therapy of PRF on DRG could induce the changes in the cell morphology. Ultrastructural changes in the axons, including abnormal membranes and morphology of mitochondria, and the disruption and disorganization of microfilaments and microtubules were observed in C- and A*δ*-fibers on electron microscopy [42]. Recently, it was reported that the analgesic effect of PRF might derive from long-term modulation of cell functions by changing gene expression. The expression of pain regulatory genes such as a proinflammatory gene returned to baseline values. Numerous reports suggested that microglia in the spinal dorsal horn were vital in pain facilitation. PRF applied on DRG in a rat model of neuropathic pain revealed that the established mechanical hypersensitivity reduced, and the activation of microglia in spinal dorsal horn was significantly attenuated [10, 11]. The findings of this study were consistent with those of previous studies. Mechanical allodynia and thermal hyperalgesia were reversed, accompanied by a significantly downregulated the expression of Iba1 which was maintained for 14 days after a single application of PRF. This demonstrated that PRF might have suppressed the activation of microglia and contributed to the nociceptive relief.

#### 5.2.2. Reversing the Increase of Microglial BDNF, PI3K, and p-ERK in the Spinal Cord of Rats with SNI

Some studies have shown that neurotrophins, especially BDNF, play an important role as pain mediators/modulators [43, 44]. BDNF is a secreted protein and part of the family of neurotrophin family. Neutrophins act on neurons to promote the survival, growth, and differentiation of new neurons and synapses. However, it has a deleterious effect on the spinal cord following nerve injury. Trang found that P2X4 receptor on the activated microglial surface promoted the synthesis and release of BDNF, which in turn accelerated central sensitization and maintained neuropathic pain [45]. Microglia-derived BDNF is a critical microglia-neuron signaling molecule that gates aberrant nociceptive processing in the spinal cord. The enhanced BDNF, which elicited nociceptive hypersensitivity, also contributed to the activation of microglia in the spinal cord in a feedforward manner, and the functional inhibition of BDNF signal reversed allodynia in rats with SNI. The upregulated expression of BDNF was detected in the spinal cord on day 21 after SNI in the present study, consistent with the previous reports, and further indicated that BDNF was highly involved in neuropathic pain.

PI3K, a lipid kinase that phosphorylates the *D*_3_ position of phosphatidylinositol lipids to produce PI(3,4,5)P_3_, acts as a membrane-embedded second messenger. Some progress has been made about the role of PI3K in the refractory pain. Plantar incision induced a time-dependent activation of PI3K in the microglia, and the inhibition of PI3K prevented pain behaviors induced by plantar incision [46]. Specific inhibitors of PI3K applied before SNI reduced the neuropathic pain behaviors induced by L5 SNL [47]. The PI3K signaling pathway was expressed in microglia and participated in bone cancer pain [48]. The treatment of bone cancer pain model in rats with PI3Kcb-specific small-interfering RNA resulted in the inhibition of pain-related behavior [49]. The expression of PI3K was assayed in the spinal cord on day 21 after SNI in the present study. PI3K was found to be significantly increased on day 21 after SNI. Therefore, the present results implied that PI3K also played an important role in neuropathic pain.

ERK, a member of the mitogen-activated protein kinase family, could transmit a great quantity of extracellular information into intracellular responses. The ERK signaling pathway in the microglia has been reported to modulate various types of pain, and the inhibition of p-ERK could alleviate the associated pain [50, 51]. The level of p-ERK in the spinal cord was found to be significantly upregulated on day 21 after SNI in the present study. These results were consistent with other reports [52] and suggested that activated ERK contributed to the development of neuropathic pain.

The microglia, BDNF, PI3K, and p-ERK were confirmed to be involved in developing neuropathic pain in present study. However, whether the release of BDNF, PI3K, and p‐ERK in the spinal cord in rats with SNI to regulate chronic pain processing is through microglia-dependent mechanism remains undetermined. In order to clarify this question, firstly, we adopted the method of triple labeling with BDNF/Iba1/DAPI, PI3K/Iba1/DAPI and p-ERK/Iba1/DAPI to prove that the localization of BDNF, PI3K, and p-ERK is in microglia. Secondly, minocycline (as microglia inhibitor) was intrathecally injected into rats with SNI at early stages; the increased levels of Iba1, BDNF, PI3K, and p-ERK in the spinal cord were all restrained. Hence, these findings indicated that BDNF, PI3K, and p-ERK might have been released in the spinal cord in a microglia-dependent manner.

Although the analgesic efficacy of PRF on DRG for neuropathic pain was exact, the mechanisms were not fully understood. The effects of PRF might, via electromagnetic fields, disrupt or somehow modulate pain signal transmission and gene expression in the treated sites and CNS. The suppression of Iba1, BDNF, PI3K, and p-ERK in the spinal cord was important in alleviating neuropathic pain in rats with SNI in the present study, and the release of BDNF, PI3K, and p-ERK in the spinal cord might have occurred in a microglia-dependent manner. Since the therapy of PRF on DRG in rats with neuropathic pain could induce pain relief by reducing microglial activation, we deduced that PRF might regulate the release of BDNF, PI3K, and p-ERK in the spinal cord to relieve neuropathic pain. Just as we assumed, the increased expression of BDNF, PI3K, and p-ERK on day 21 was simultaneously downregulated for 6 min PRF therapy after SNI. So, these results revealed that PRF therapy on DRG might attenuate neuropathic pain by reducing the release of BDNF, PI3K, and p-ERK in the spinal cord in microglia-dependent way.

## 6. Limitations

This study had some limitations. Firstly, only the short-term effectiveness of PRF on DRG was explored. Secondly, *in vivo* field potential recording in PRF-treated rats was absent. Thirdly, only one time point was selected to assay the levels of Iba1, BDNF, PI3K, and p-ERK. Fourthly, the relationship among BDNF, PI3K, and p-ERK was not investigated. More detailed research about PRF on the DRG to ease the neuropathic pain is needed.

## 7. Conclusions

The application of PRF on DRG could reverse SNI-induced neuropathic pain after the treatment period. The mechanisms underlying this treatment might be suppressed microglia and downregulated levels of BDNF, PI3K, and p-ERK in the spinal cord in microglia-dependent way. It supported PRF treatment as a valuable intervention for chronic neuropathic pain.

## Data Availability

The data used to support the findings of this study are available from the corresponding author upon request.

## References

[1] Obata N., Mizobuchi S., Itano Y. (2011). Decoy strategy targeting the brain-derived neurotrophic factor exon I to attenuate tactile allodynia in the neuropathic pain model of rats. *Biochemical and Biophysical Research Communications*.

[2] Omori S., Isose S., Misawa S. (2017). Pain-related evoked potentials after intraepidermal electrical stimulation to A*δ* and C fibers in patients with neuropathic pain. *Neuroscience Research*.

[3] Biggs J. E., Lu V. B., Stebbing M. J. (2010). Is BDNF sufficient for information transfer between microglia and dorsal horn neurons during the onset of central sensitization?. *Molecular Pain*.

[4] Djouhri L. (2016). L5 spinal nerve axotomy induces sensitization of cutaneous L4 A*β*-nociceptive dorsal root ganglion neurons in the rat *in vivo*. *Neuroscience Letters*.

[5] Nijs J., Meeus M., Versijpt J. (2015). Brain-derived neurotrophic factor as a driving force behind neuroplasticity in neuropathic and central sensitization pain: a new therapeutic target?. *Expert Opinion on Therapeutic Targets*.

[6] Truini A., Garcia-Larrea L., Cruccu G. (2013). Reappraising neuropathic pain in humans-how symptoms help disclose mechanisms. *Nature Reviews Neurology*.

[7] Udina E., Cobianchi S., Allodi I., Navarro X. (2011). Effects of activity-dependent strategies on regeneration and plasticity after peripheral nerve injuries. *Annals of Anatomy—Anatomischer Anzeiger*.

[8] Halim W., van der Weegen W., Lim T., Wullems J. A., Vissers K. C. (2017). Percutaneous cervical nucleoplasty vs. pulsed radio frequency of the dorsal root ganglion in patients with contained cervical disk herniation; a prospective, randomized controlled trial. *Pain Practice*.

[9] Kim K., Jo D., Kim E. (2017). Pulsed radiofrequency to the dorsal root ganglion in acute herpes zoster and postherpetic neuralgia. *Pain Physician*.

[10] Park H.-W., Ahn S.-H., Son J.-Y. (2012). Pulsed radiofrequency application reduced mechanical hypersensitivity and microglial expression in neuropathic pain model. *Pain Medicine*.

[11] Cho H. K., Cho Y. W., Kim E. H., Sluijter M. E., Hwang S. J., Ahn S. H. (2013). Changes in pain behavior and glial activation in the spinal dorsal horn after pulsed radiofrequency current administration to the dorsal root ganglion in a rat model of lumbar disc herniation. *Journal of Neurosurgery: Spine*.

[12] Perry V. H., Holmes C. (2014). Microglial priming in neurodegenerative disease. *Nature Reviews Neurology*.

[13] Masuda T., Tsuda M., Yoshinaga R. (2012). IRF8 is a critical transcription factor for transforming microglia into a reactive phenotype. *Cell Reports*.

[14] Meacham K., Shepherd A., Mohapatra D. P., Haroutounian S. (2017). Neuropathic pain: central vs. peripheral mechanisms. *Current Pain and Headache Reports*.

[15] Trang T., Beggs S., Salter M. W. (2011). Brain-derived neurotrophic factor from microglia: a molecular substrate for neuropathic pain. *Neuron Glia Biology*.

[16] Sikandar S., Minett M. S., Millet Q. (2018). Brain-derived neurotrophic factor derived from sensory neurons plays a critical role in chronic pain. *Brain*.

[17] Liu M., Kay J. C., Shen S., Qiao L.-Y. (2015). Endogenous BDNF augments NMDA receptor phosphorylation in the spinal cord via PLC*γ*, PKC, and PI3K/Akt pathways during colitis. *Journal of Neuroinflammation*.

[18] Odaka H., Numakawa T., Yoshimura A. (2016). Chronic glucocorticoid exposure suppressed the differentiation and survival of embryonic neural stem/progenitor cells: possible involvement of ERK and PI3K/Akt signaling in the neuronal differentiation. *Neuroscience Research*.

[19] Pezet S., Marchand F., D’Mello R. (2008). Phosphatidylinositol 3-kinase is a key mediator of central sensitization in painful inflammatory conditions. *Journal of Neuroscience*.

[20] Shibuta K., Suzuki I., Shinoda M. (2012). Organization of hyperactive microglial cells in trigeminal spinal subnucleus caudalis and upper cervical spinal cord associated with orofacial neuropathic pain. *Brain Research*.

[21] Decosterd I., Woolf C. J. (2000). Spared nerve injury: an animal model of persistent peripheral neuropathic pain. *Pain*.

[22] Li X., Yang H., Ouyang Q. (2016). Enhanced RAGE expression in the dorsal root ganglion may contribute to neuropathic pain induced by spinal nerve ligation in rats. *Pain Medicine*.

[23] Chaplan S. R., Bach F. W., Pogrel J. W., Chung J. M., Yaksh T. L. (1994). Quantitative assessment of tactile allodynia in the rat paw. *Journal of Neuroscience Methods*.

[24] Hargreaves K., Dubner R., Brown F., Flores C., Joris J. (1988). A new and sensitive method for measuring thermal nociception in cutaneous hyperalgesia. *Pain*.

[25] Ikeda H., Kiritoshi T., Murase K. (2012). Contribution of microglia and astrocytes to the central sensitization, inflammatory and neuropathic pain in the juvenile rat. *Molecular Pain*.

[26] Liem L., van Dongen E., Huygen F. J., Staats P., Kramer J. (2016). The dorsal root ganglion as a therapeutic target for chronic pain. *Regional Anesthesia and Pain Medicine*.

[27] Arai Y.-C. P., Nishihara M., Yamamoto Y. (2015). Dorsal root ganglion pulsed radiofrequency for the management of intractable vertebral metastatic pain: a case series. *Pain Medicine*.

[28] Van Boxem K., de Meij N., Kessels A., Van Kleef M., Van Zundert J. (2015). Pulsed radiofrequency for chronic intractable lumbosacral radicular pain: a six-month cohort study. *Pain Medicine*.

[29] You J., Gao J., Chen P. (2013). Changes of basic fibroblast growth factor expression in the spinal cord of rats with spared nerve injury of the sciatic nerve. *Nan Fang Yi Ke Da Xue Xue Bao*.

[30] Lin M.-L., Lin W.-T., Huang R.-Y. (2014). Pulsed radiofrequency inhibited activation of spinal mitogen-activated protein kinases and ameliorated early neuropathic pain in rats. *European Journal of Pain*.

[31] Chen K.-H., Yang C.-H., Juang S.-E. (2014). Pulsed radiofrequency reduced complete Freund’s adjuvant-induced mechanical hyperalgesia via the spinal c-Jun N-terminal kinase pathway. *Cellular and Molecular Neurobiology*.

[32] Yang C.-H., Chen K.-H., Huang H.-W., Sheen-Chen S.-M., Lin C.-R. (2013). Pulsed radiofrequency treatment attenuates increases in spinal excitatory amino acid release in rats with adjuvant-induced mechanical allodynia. *NeuroReport*.

[33] Perret D. M., Kim D. S., Li K. W. (2011). Application of pulsed radiofrequency currents to rat dorsal root ganglia modulates nerve injury-induced tactile allodynia. *Anesthesia & Analgesia*.

[34] Tanaka N., Yamaga M., Tateyama S., Uno T., Tsuneyoshi I., Takasaki M. (2010). The effect of pulsed radiofrequency current on mechanical allodynia induced with resiniferatoxin in rats. *Anesthesia & Analgesia*.

[35] Liu Y., Feng Y., Zhang T. (2015). Pulsed radiofrequency treatment enhances dorsal root ganglion expression of hyperpolarization-activated cyclic nucleotide-gated channels in a rat model of neuropathic pain. *Journal of Molecular Neuroscience*.

[36] Ji R. R., Suter M. R. (2007). p38 MAPK, microglial signaling, and neuropathic pain. *Molecular Pain*.

[37] Coull J. A. M., Beggs S., Boudreau D. (2005). BDNF from microglia causes the shift in neuronal anion gradient underlying neuropathic pain. *Nature*.

[38] Mika J., Zychowska M., Popiolek-Barczyk K., Rojewska E., Przewlocka B. (2013). Importance of glial activation in neuropathic pain. *European Journal of Pharmacology*.

[39] Hu F., Zhang H.-H., Yang B.-X. (2015). Cdk5 contributes to inflammation-induced thermal hyperalgesia mediated by the p38 MAPK pathway in microglia. *Brain Research*.

[40] Terayama R., Omura S., Fujisawa N., Yamaai T., Ichikawa H., Sugimoto T. (2008). Activation of microglia and p38 mitogen-activated protein kinase in the dorsal column nucleus contributes to tactile allodynia following peripheral nerve injury. *Neuroscience*.

[41] Raghavendra V., Tanga F., DeLeo J. A. (2003). Inhibition of microglial activation attenuates the development but not existing hypersensitivity in a rat model of neuropathy. *Journal of Pharmacology and Experimental Therapeutics*.

[42] Erdine S., Bilir A., Cosman E. R., Cosman E. R. (2009). Ultrastructural changes in axons following exposure to pulsed radiofrequency fields. *Pain Practice*.

[43] Smith P. A. (2014). BDNF: no gain without pain?. *Neuroscience*.

[44] Miao J., Ding M., Zhang A. (2012). Pleiotrophin promotes microglia proliferation and secretion of neurotrophic factors by activating extracellular signal-regulated kinase 1/2 pathway. *Neuroscience Research*.

[45] Trang T., Beggs S., Wan X., Salter M. W. (2009). P2X4-receptor-mediated synthesis and release of brain-derived neurotrophic factor in microglia is dependent on calcium and p38-mitogen-activated protein kinase activation. *Journal of Neuroscience*.

[46] Xu B., Guan X.-H., Yu J.-X. (2014). Activation of spinal phosphatidylinositol 3-kinase/protein kinase B mediates pain behavior induced by plantar incision in mice. *Experimental Neurology*.

[47] Xu J.-T., Tu H.-Y., Xin W.-J., Liu X.-G., Zhang G.-H., Zhai C.-H. (2007). Activation of phosphatidylinositol 3-kinase and protein kinase B/Akt in dorsal root ganglia and spinal cord contributes to the neuropathic pain induced by spinal nerve ligation in rats. *Experimental Neurology*.

[48] Jin D., Yang J.-P., Hu J.-H., Wang L.-N., Zuo J.-L. (2015). MCP-1 stimulates spinal microglia via PI3K/Akt pathway in bone cancer pain. *Brain Research*.

[49] Huang H. J., Zhang M. (2014). Downregulation of PI3Kcb utilizing adenovirus-mediated transfer of siRNA attenuates bone cancer pain. *International Journal of Clinical and Experimental Pathology*.

[50] Zhang X., Zhang H., Shao H., Xue Q., Yu B. (2014). ERK MAP kinase activation in spinal cord regulates phosphorylation of Cdk5 at serine 159 and contributes to peripheral inflammation induced pain/hypersensitivity. *PLoS One*.

[51] Guo Y.-J., Shi X.-D., Fu D., Yang Y., Wang Y.-P., Dai R.-P. (2013). Analgesic effects of the COX-2 inhibitor parecoxib on surgical pain through suppression of spinal ERK signaling. *Experimental and Therapeutic Medicine*.

[52] Calvo M., Zhu N., Grist J., Ma Z., Loeb J. A., Bennett D. L. H. (2011). Following nerve injury neuregulin-1 drives microglial proliferation and neuropathic pain via the MEK/ERK pathway. *Glia*.

